# Crystal structure of (2-hy­droxy-5-methyl­phen­yl)(3-methyl-1-phenyl-1*H*-pyrazolo­[3,4-*b*]pyridin-5-yl)methanone

**DOI:** 10.1107/S2056989015011597

**Published:** 2015-06-24

**Authors:** Rajamani Raja, Nataraj Poomathi, Paramasivam T. Perumal, A. SubbiahPandi

**Affiliations:** a Department of Physics, Presidency College (Autonomous), Chennai 600 005, India; b Organic Chemistry Division, CSIR Central Leather Research Institute, Adyar, Chennai 600 020, India

**Keywords:** crystal structure, pyrazoles, propenones, pyrazolo­pyridine, intra­molecular hydrogen bonding, π–π inter­actions, C—H⋯π inter­actions

## Abstract

In the title compound, C_21_H_17_N_3_O_2_, the 2-hy­droxy-5-methyl­phenyl ring and the phenyl ring are inclined to the mean plane of the pyrazolo­pyridine moiety (r.m.s. deviation = 0.013 Å) by 52.89 (9) and 19.63 (8)°, respectively, and to each other by 42.83 (11)°. In the mol­ecule, there are intra­molecular O—H⋯O and C—H⋯N hydrogen bonds, both enclosing an *S*(6) ring motif. In the crystal, mol­ecules stack along the *c*-axis direction, forming columns within which there are a number of π–π inter­actions [the inter-centroid distances vary from 3.5278 (10) to 3.8625 (10) Å]. The columns are linked by C—H⋯π inter­actions, forming slabs parallel to (100).

## Related literature   

For some details of the biological activity of pyrazole derivatives, see: Burger & Iorio (1979 ▸, 1980 ▸); Kalluraya & Ramesh (2001 ▸); Windholz (2003 ▸). For the anti­bacterial activity of propenones, see: Holla *et al.* (1994 ▸). For details of the pyrazole moiety found in blockbuster drugs, see: Penning *et al.* (1997 ▸) for celecobix; Terrett *et al.* (1996 ▸) for sildenafil; Seltzman *et al.* (1995 ▸) for rimonabant.
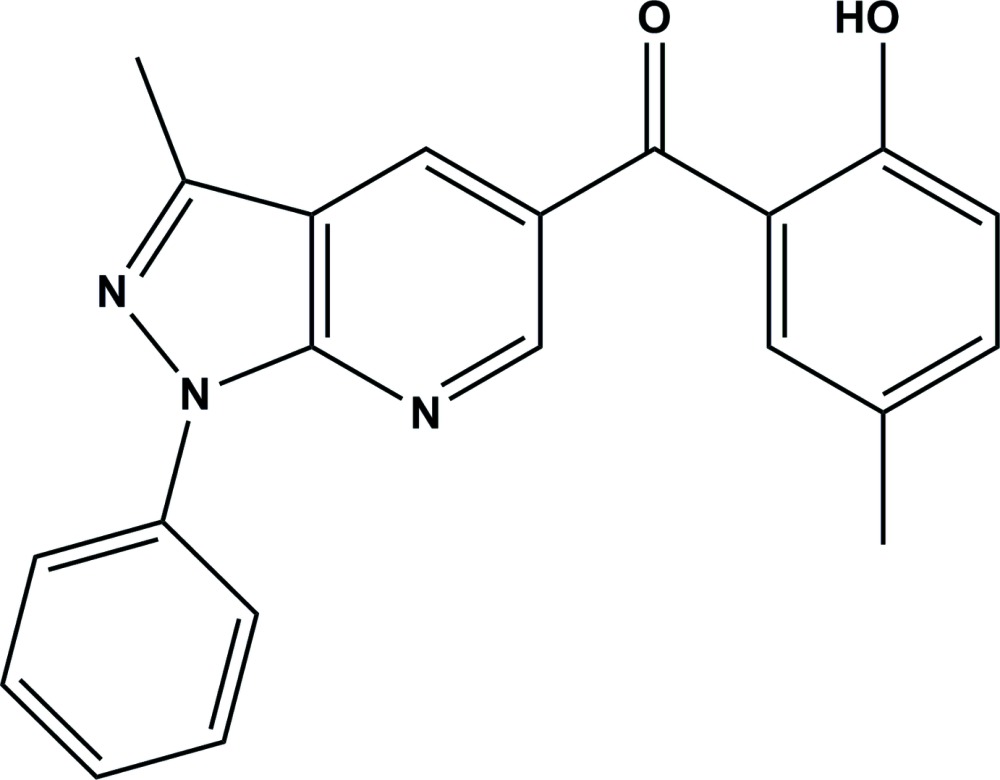


## Experimental   

### Crystal data  

C_21_H_17_N_3_O_2_
*M* *_r_* = 343.38
Monoclinic, 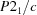

*a* = 14.7164 (7) Å
*b* = 16.7306 (9) Å
*c* = 7.0733 (3) Å
β = 94.857 (2)°
*V* = 1735.29 (14) Å^3^
*Z* = 4
Mo *K*α radiation
μ = 0.09 mm^−1^
*T* = 293 K
0.25 × 0.20 × 0.20 mm

### Data collection  

Bruker SMART APEXII CCD diffractometer
Absorption correction: multi-scan (*SADABS*; Bruker, 2008 ▸) *T*_min_ = 0.979, *T*_max_ = 0.983
10536 measured reflections
3055 independent reflections
2001 reflections with *I* > 2σ(*I*)
*R*_int_ = 0.036

### Refinement  

*R*[*F* ^2^ > 2σ(*F* ^2^)] = 0.045
*wR*(*F* ^2^) = 0.125
*S* = 1.04
3055 reflections
237 parameters
H-atom parameters constrained
Δρ_max_ = 0.20 e Å^−3^
Δρ_min_ = −0.18 e Å^−3^

### 

Data collection: *APEX2* (Bruker, 2008 ▸); cell refinement: *SAINT* (Bruker, 2008 ▸); data reduction: *SAINT*; program(s) used to solve structure: *SHELXS97* (Sheldrick, 2008 ▸); program(s) used to refine structure: *SHELXL97* (Sheldrick, 2008 ▸); molecular graphics: *ORTEP-3 for Windows* (Farrugia, 2012 ▸); software used to prepare material for publication: *SHELXL97* and *PLATON* (Spek, 2009 ▸).

## Supplementary Material

Crystal structure: contains datablock(s) global, I. DOI: 10.1107/S2056989015011597/su5154sup1.cif

Structure factors: contains datablock(s) I. DOI: 10.1107/S2056989015011597/su5154Isup2.hkl

Supporting information file. DOI: 10.1107/S2056989015011597/su5154Isup3.cml

**Fig. 1.**
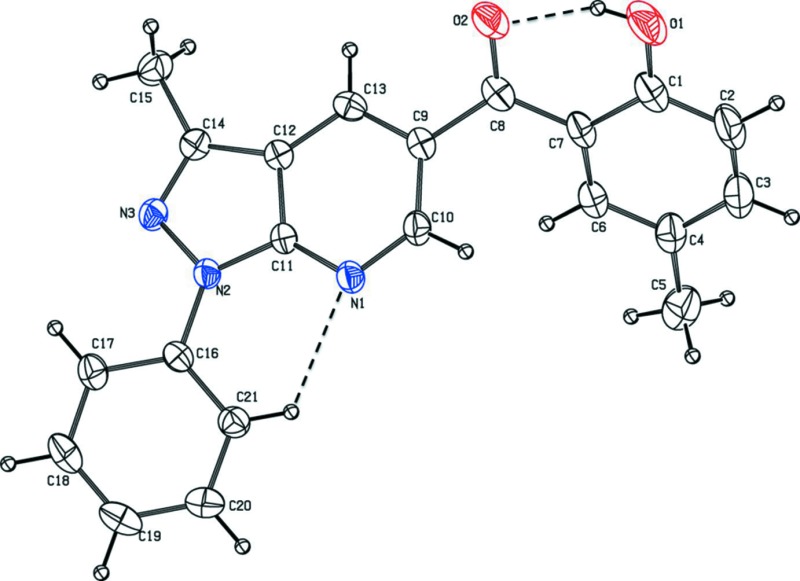
The molecular structure of the title compound, with atom labelling. Displacement ellipsoids are drawn at the 30% probability level. Intramolecular hydrogen bonds are shown as dashed lines (see Table 1 for details)

**Fig. 2.**
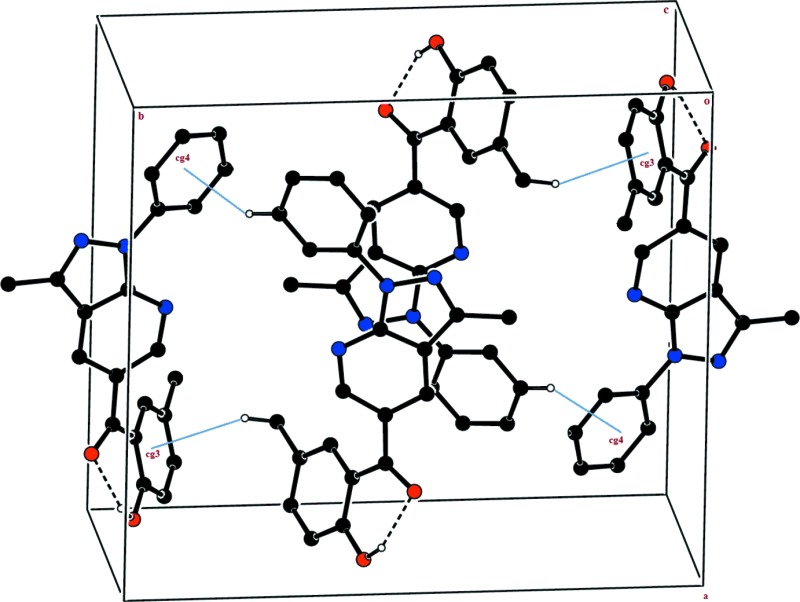
The crystal packing of the title compound, viewed along the c axis. The O—H···O and C—H···π interactions are shown as dashed lines (see Table 1 for details).

. DOI: 10.1107/S2056989015011597/su5154fig1.tif
The mol­ecular structure of the title compound, with atom labelling. Displacement ellipsoids are drawn at the 30% probability level. Intra­molecular hydrogen bonds are shown as dashed lines (see Table 1 for details)

c . DOI: 10.1107/S2056989015011597/su5154fig2.tif
The crystal packing of the title compound, viewed along the *c* axis. The O—H⋯O and C—H⋯π inter­actions are shown as dashed lines (see Table 1 for details).

CCDC reference: 1406889

Additional supporting information:  crystallographic information; 3D view; checkCIF report

## Figures and Tables

**Table 1:** Hydrogen-bond geometry (, ) *Cg*3 and *Cg*4 are the centroids of rings C1C4/C6/C7 and C16C21, respectively.

*D*H*A*	*D*H	H*A*	*D* *A*	*D*H*A*
O1H1O2	0.82	1.91	2.613(2)	143
C21H21N1	0.93	2.41	3.019(3)	123
C5H5*A* *Cg*3^i^	0.96	2.97	3.703(3)	134
C20H20*Cg*4^i^	0.93	2.80	3.608(2)	146

Symmetry code: (i) 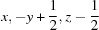.

## supplementary crystallographic information

### S1. Comments

Pyrazole derivatives are reported to possess varied biological activities such as anti-inflammatory (Windholz, 2003), analgesic (Windholz, 2003), hypoglysemic, seditive (Burger *et al.*, 1979), hypnotic (Burger *et al.*, 1980), anti­fungal and anti­bacterial (Kalluraya & Ramesh, 2001) activities. Propenones are also found to show good anti­bacterial activity (Holla *et al.*, 1994). The pyrazole moiety is found in blockbuster drugs such as celecobix (Penning *et al.*, 1997), sildenafil (Terrett *et al.*, 1996) and rimonabant (Seltzmann *et al.*, 1995).

The molecular structure of the title molecule is shown in Fig. 1. The 2-hy­droxy-5-methyl­phenyl ring (C1—C4/C6/C7) and the phenyl ring (C16—C21) are inclined to the mean plane of the pyrazolo­pyridine moiety (N1—N3/C9—C14; r.m.s. deviation = 0.013 Å) by 52.89 (9) and 19.63 (8) °, respectively, and to each other by 42.83 (11) °. The molecular conformation is partly determined by the intra­molecular O—H···O hydrogen bond with an S(6) ring motif, and a C—H···N short contact enclosing a second S(6) ring motif (Table 1 and Fig. 1).

In the crystal, the molecules stack along the *c* axis direction forming columns, within which there are a number of π–π inter­actions [Cg1···Cg1^i^ = 3.7660 (10) Å, inter­planar distance = 3.4748 (7) Å, slippage = 1.452 Å; Cg1··· Cg1^ii^ = 3.5278 (10) Å, inter­planar distance = 3.4477 (7) Å, slippage = 0.747 Å; Cg1···Cg2^i^ = 3.6162 (10) Å; Cg1···Cg2^ii^ = 3.8625 (10) Å; Cg1 and Cg2 are the centroids of rings N2/N3/C11—C14 and N1/C9—C13, respectively; symmetry codes: (i) -x + 1, -y, -z; (ii) -x + 1, -y, -z + 1]. The columns are linked by C—H···π inter­actions (Table 1 and Fig. 2) forming slabs parallel to (100).

### S2. Synthesis and crystallization

To a mixture of 3-formyl­chromone and 5-amino-3-methyl-1-phenyl pyrazole in ethanol, was added a catalytic amount of In(OTf)_3_ and the resulting mixture was refluxed for ca. 20 min. The precipitate formed was filtered and dried under vacuum to afford the pure title product (yield: 87%). It was recrystallized from ethanol and DMSO-D_6_ by slow evaporation over 48 h, giving colourless block-like crystals.

### S3. Refinement

Crystal data, data collection and structure refinement details are summarized in Table 2. The O- and C-bound H atoms were positioned geometrically and allowed to ride on their parent atoms: O—H = 0.82 Å, C–H = 0.93–0.96 Å with *U*_iso_(H) = 1.5*U*_eq_(O,C) for the hydroxyl and methyl H atoms and 1.2*U*_eq_(C) for other H atoms.

### Crystal data

**Table:**

C_21_H_17_N_3_O_2_	*F*(000) = 720
*M**_r_* = 343.38	*D*_x_ = 1.314 Mg m^−^^3^
Monoclinic, *P*2_1_/*c*	Mo *K*α radiation, λ = 0.71073 Å
Hall symbol: -P 2ybc	Cell parameters from 2001 reflections
*a* = 14.7164 (7) Å	θ = 1.4–25.0°
*b* = 16.7306 (9) Å	µ = 0.09 mm^−^^1^
*c* = 7.0733 (3) Å	*T* = 293 K
β = 94.857 (2)°	Block, colourless
*V* = 1735.29 (14) Å^3^	0.25 × 0.20 × 0.20 mm
*Z* = 4	

### Data collection

**Table:**

Bruker SMART APEXII CCD diffractometer	3055 independent reflections
Radiation source: fine-focus sealed tube	2001 reflections with *I* > 2σ(*I*)
Graphite monochromator	*R*_int_ = 0.036
ω and φ scans	θ_max_ = 25.0°, θ_min_ = 1.4°
Absorption correction: multi-scan (*SADABS*; Bruker, 2008)	*h* = −17→17
*T*_min_ = 0.979, *T*_max_ = 0.983	*k* = −19→19
10536 measured reflections	*l* = −5→8

### Refinement

**Table:**

Refinement on *F*^2^	Primary atom site location: structure-invariant direct methods
Least-squares matrix: full	Secondary atom site location: difference Fourier map
*R*[*F*^2^ > 2σ(*F*^2^)] = 0.045	Hydrogen site location: inferred from neighbouring sites
*wR*(*F*^2^) = 0.125	H-atom parameters constrained
*S* = 1.04	*w* = 1/[σ^2^(*F*_o_^2^) + (0.0597*P*)^2^ + 0.1547*P*] where *P* = (*F*_o_^2^ + 2*F*_c_^2^)/3
3055 reflections	(Δ/σ)_max_ < 0.001
237 parameters	Δρ_max_ = 0.20 e Å^−^^3^
0 restraints	Δρ_min_ = −0.18 e Å^−^^3^

### Special details

**Table:**

Geometry. All e.s.d.'s (except the e.s.d. in the dihedral angle between two l.s. planes) are estimated using the full covariance matrix. The cell e.s.d.'s are taken into account individually in the estimation of e.s.d.'s in distances, angles and torsion angles; correlations between e.s.d.'s in cell parameters are only used when they are defined by crystal symmetry. An approximate (isotropic) treatment of cell e.s.d.'s is used for estimating e.s.d.'s involving l.s. planes.
Refinement. Refinement of *F*^2^ against ALL reflections. The weighted *R*-factor *wR* and goodness of fit *S* are based on *F*^2^, conventional *R*-factors *R* are based on *F*, with *F* set to zero for negative *F*^2^. The threshold expression of *F*^2^ > σ(*F*^2^) is used only for calculating *R*-factors(gt) *etc*. and is not relevant to the choice of reflections for refinement. *R*-factors based on *F*^2^ are statistically about twice as large as those based on *F*, and *R*- factors based on ALL data will be even larger.

### Fractional atomic coordinates and isotropic or equivalent isotropic displacement parameters (Å^2^)

**Table:**

	*x*	*y*	*z*	*U*_iso_*/*U*_eq_	
C1	0.05588 (15)	0.09390 (16)	0.0431 (4)	0.0621 (7)	
C2	0.00532 (16)	0.13878 (17)	−0.0938 (4)	0.0746 (8)	
H2	−0.0552	0.1509	−0.0781	0.090*	
C3	0.04416 (17)	0.16526 (16)	−0.2520 (4)	0.0724 (8)	
H3	0.0090	0.1950	−0.3420	0.087*	
C4	0.13449 (15)	0.14903 (14)	−0.2816 (3)	0.0559 (6)	
C5	0.17618 (19)	0.17799 (17)	−0.4560 (4)	0.0787 (8)	
H5A	0.1872	0.2344	−0.4458	0.118*	
H5B	0.1352	0.1674	−0.5660	0.118*	
H5C	0.2328	0.1506	−0.4675	0.118*	
C6	0.18475 (14)	0.10574 (13)	−0.1436 (3)	0.0491 (6)	
H6	0.2454	0.0945	−0.1602	0.059*	
C7	0.14798 (13)	0.07811 (13)	0.0206 (3)	0.0482 (6)	
C8	0.20198 (14)	0.03024 (15)	0.1632 (3)	0.0517 (6)	
C9	0.30348 (13)	0.03373 (13)	0.1803 (3)	0.0420 (5)	
C10	0.35019 (13)	0.10607 (14)	0.1603 (3)	0.0451 (5)	
H10	0.3150	0.1511	0.1289	0.054*	
C11	0.48557 (12)	0.04804 (12)	0.2241 (2)	0.0358 (5)	
C12	0.44789 (12)	−0.02748 (12)	0.2511 (2)	0.0364 (5)	
C13	0.35323 (13)	−0.03413 (13)	0.2305 (3)	0.0426 (5)	
H13	0.3245	−0.0826	0.2500	0.051*	
C14	0.52392 (13)	−0.07907 (13)	0.2957 (3)	0.0409 (5)	
C15	0.52419 (16)	−0.16642 (14)	0.3369 (3)	0.0585 (6)	
H15A	0.5858	−0.1858	0.3471	0.088*	
H15B	0.4895	−0.1940	0.2360	0.088*	
H15C	0.4975	−0.1757	0.4542	0.088*	
C16	0.64979 (12)	0.09625 (12)	0.2357 (2)	0.0376 (5)	
C17	0.73654 (13)	0.07915 (14)	0.3181 (3)	0.0484 (6)	
H17	0.7475	0.0326	0.3881	0.058*	
C18	0.80646 (14)	0.13251 (17)	0.2945 (3)	0.0618 (7)	
H18	0.8650	0.1211	0.3478	0.074*	
C19	0.79085 (16)	0.20215 (17)	0.1933 (3)	0.0639 (7)	
H19	0.8384	0.2375	0.1783	0.077*	
C20	0.70447 (15)	0.21893 (15)	0.1146 (3)	0.0548 (6)	
H20	0.6935	0.2662	0.0473	0.066*	
C21	0.63382 (14)	0.16638 (13)	0.1345 (3)	0.0448 (5)	
H21	0.5756	0.1780	0.0800	0.054*	
N1	0.44001 (10)	0.11614 (10)	0.1820 (2)	0.0426 (4)	
N2	0.57850 (10)	0.03981 (10)	0.2511 (2)	0.0378 (4)	
N3	0.60079 (11)	−0.03890 (10)	0.2955 (2)	0.0421 (4)	
O1	0.01363 (10)	0.06702 (12)	0.1927 (3)	0.0870 (6)	
H1	0.0497	0.0411	0.2629	0.130*	
O2	0.16431 (10)	−0.01281 (12)	0.2757 (2)	0.0770 (6)	

### Atomic displacement parameters (Å^2^)

**Table:**

	*U*^11^	*U*^22^	*U*^33^	*U*^12^	*U*^13^	*U*^23^
C1	0.0363 (13)	0.0691 (19)	0.0801 (16)	−0.0013 (12)	0.0012 (12)	−0.0142 (14)
C2	0.0360 (13)	0.078 (2)	0.108 (2)	0.0116 (13)	−0.0070 (14)	−0.0114 (17)
C3	0.0540 (16)	0.0674 (19)	0.0910 (19)	0.0122 (14)	−0.0217 (14)	−0.0046 (16)
C4	0.0514 (14)	0.0499 (16)	0.0636 (14)	0.0071 (12)	−0.0111 (11)	−0.0101 (12)
C5	0.0879 (19)	0.076 (2)	0.0695 (16)	0.0080 (16)	−0.0083 (14)	0.0057 (14)
C6	0.0362 (11)	0.0506 (15)	0.0591 (13)	0.0039 (10)	−0.0035 (10)	−0.0120 (11)
C7	0.0296 (11)	0.0521 (15)	0.0622 (13)	0.0028 (10)	−0.0007 (10)	−0.0120 (11)
C8	0.0384 (12)	0.0626 (17)	0.0542 (13)	−0.0040 (11)	0.0044 (10)	−0.0059 (12)
C9	0.0336 (11)	0.0484 (14)	0.0438 (11)	0.0006 (10)	0.0012 (8)	−0.0025 (10)
C10	0.0368 (12)	0.0459 (14)	0.0516 (12)	0.0073 (10)	−0.0020 (9)	−0.0009 (10)
C11	0.0317 (10)	0.0394 (13)	0.0361 (10)	0.0031 (9)	0.0016 (8)	−0.0025 (9)
C12	0.0366 (11)	0.0377 (13)	0.0348 (9)	0.0001 (9)	0.0025 (8)	−0.0048 (9)
C13	0.0425 (12)	0.0451 (14)	0.0407 (10)	−0.0082 (10)	0.0062 (9)	−0.0048 (9)
C14	0.0447 (12)	0.0382 (13)	0.0396 (10)	0.0015 (10)	0.0030 (9)	−0.0029 (9)
C15	0.0648 (15)	0.0400 (15)	0.0693 (14)	0.0025 (12)	−0.0020 (12)	0.0037 (11)
C16	0.0337 (11)	0.0435 (14)	0.0363 (10)	−0.0009 (9)	0.0071 (8)	−0.0068 (9)
C17	0.0373 (12)	0.0518 (15)	0.0558 (12)	0.0066 (11)	0.0023 (9)	−0.0054 (11)
C18	0.0319 (12)	0.076 (2)	0.0775 (16)	−0.0011 (12)	0.0064 (11)	−0.0127 (15)
C19	0.0485 (15)	0.072 (2)	0.0747 (16)	−0.0190 (13)	0.0219 (12)	−0.0100 (14)
C20	0.0579 (15)	0.0539 (16)	0.0541 (12)	−0.0116 (12)	0.0134 (11)	0.0017 (11)
C21	0.0432 (12)	0.0477 (15)	0.0432 (11)	−0.0032 (10)	0.0026 (9)	0.0001 (10)
N1	0.0340 (9)	0.0412 (11)	0.0520 (10)	0.0031 (8)	0.0003 (7)	−0.0012 (8)
N2	0.0324 (9)	0.0364 (11)	0.0443 (9)	0.0032 (8)	0.0013 (7)	0.0005 (8)
N3	0.0403 (10)	0.0367 (11)	0.0490 (9)	0.0066 (8)	0.0020 (7)	0.0006 (8)
O1	0.0393 (10)	0.1199 (18)	0.1043 (14)	−0.0003 (10)	0.0207 (9)	0.0018 (12)
O2	0.0431 (9)	0.1069 (16)	0.0816 (11)	−0.0112 (10)	0.0087 (8)	0.0238 (11)

### Geometric parameters (Å, º)

**Table:**

C1—O1	1.349 (3)	C11—C12	1.399 (3)
C1—C2	1.391 (3)	C12—C13	1.393 (3)
C1—C7	1.403 (3)	C12—C14	1.427 (3)
C2—C3	1.372 (4)	C13—H13	0.9300
C2—H2	0.9300	C14—N3	1.316 (2)
C3—C4	1.390 (3)	C14—C15	1.490 (3)
C3—H3	0.9300	C15—H15A	0.9600
C4—C6	1.379 (3)	C15—H15B	0.9600
C4—C5	1.504 (3)	C15—H15C	0.9600
C5—H5A	0.9600	C16—C21	1.384 (3)
C5—H5B	0.9600	C16—C17	1.388 (3)
C5—H5C	0.9600	C16—N2	1.423 (2)
C6—C7	1.401 (3)	C17—C18	1.383 (3)
C6—H6	0.9300	C17—H17	0.9300
C7—C8	1.468 (3)	C18—C19	1.376 (3)
C8—O2	1.239 (3)	C18—H18	0.9300
C8—C9	1.489 (3)	C19—C20	1.373 (3)
C9—C13	1.381 (3)	C19—H19	0.9300
C9—C10	1.405 (3)	C20—C21	1.378 (3)
C10—N1	1.328 (2)	C20—H20	0.9300
C10—H10	0.9300	C21—H21	0.9300
C11—N1	1.343 (2)	N2—N3	1.387 (2)
C11—N2	1.372 (2)	O1—H1	0.8200
			
O1—C1—C2	118.1 (2)	C11—C12—C14	105.31 (17)
O1—C1—C7	122.7 (2)	C9—C13—C12	117.71 (19)
C2—C1—C7	119.2 (2)	C9—C13—H13	121.1
C3—C2—C1	120.5 (2)	C12—C13—H13	121.1
C3—C2—H2	119.8	N3—C14—C12	110.41 (18)
C1—C2—H2	119.8	N3—C14—C15	120.87 (19)
C2—C3—C4	122.0 (2)	C12—C14—C15	128.72 (19)
C2—C3—H3	119.0	C14—C15—H15A	109.5
C4—C3—H3	119.0	C14—C15—H15B	109.5
C6—C4—C3	117.3 (2)	H15A—C15—H15B	109.5
C6—C4—C5	121.2 (2)	C14—C15—H15C	109.5
C3—C4—C5	121.5 (2)	H15A—C15—H15C	109.5
C4—C5—H5A	109.5	H15B—C15—H15C	109.5
C4—C5—H5B	109.5	C21—C16—C17	120.13 (19)
H5A—C5—H5B	109.5	C21—C16—N2	120.56 (16)
C4—C5—H5C	109.5	C17—C16—N2	119.28 (18)
H5A—C5—H5C	109.5	C18—C17—C16	118.9 (2)
H5B—C5—H5C	109.5	C18—C17—H17	120.5
C4—C6—C7	122.6 (2)	C16—C17—H17	120.5
C4—C6—H6	118.7	C19—C18—C17	121.1 (2)
C7—C6—H6	118.7	C19—C18—H18	119.5
C6—C7—C1	118.5 (2)	C17—C18—H18	119.5
C6—C7—C8	121.67 (18)	C20—C19—C18	119.5 (2)
C1—C7—C8	119.8 (2)	C20—C19—H19	120.3
O2—C8—C7	120.86 (19)	C18—C19—H19	120.3
O2—C8—C9	118.09 (19)	C19—C20—C21	120.6 (2)
C7—C8—C9	121.03 (19)	C19—C20—H20	119.7
C13—C9—C10	118.74 (18)	C21—C20—H20	119.7
C13—C9—C8	119.7 (2)	C20—C21—C16	119.81 (19)
C10—C9—C8	121.43 (19)	C20—C21—H21	120.1
N1—C10—C9	126.03 (19)	C16—C21—H21	120.1
N1—C10—H10	117.0	C10—N1—C11	113.03 (18)
C9—C10—H10	117.0	C11—N2—N3	109.93 (16)
N1—C11—N2	126.11 (18)	C11—N2—C16	130.97 (17)
N1—C11—C12	126.89 (17)	N3—N2—C16	119.08 (15)
N2—C11—C12	107.00 (17)	C14—N3—N2	107.36 (15)
C13—C12—C11	117.53 (18)	C1—O1—H1	109.5
C13—C12—C14	137.2 (2)		
			
O1—C1—C2—C3	−177.8 (2)	C11—C12—C13—C9	1.6 (2)
C7—C1—C2—C3	2.0 (4)	C14—C12—C13—C9	−178.63 (19)
C1—C2—C3—C4	−0.3 (4)	C13—C12—C14—N3	−179.9 (2)
C2—C3—C4—C6	−0.9 (4)	C11—C12—C14—N3	−0.1 (2)
C2—C3—C4—C5	179.7 (2)	C13—C12—C14—C15	0.7 (4)
C3—C4—C6—C7	0.3 (3)	C11—C12—C14—C15	−179.57 (19)
C5—C4—C6—C7	179.8 (2)	C21—C16—C17—C18	−1.1 (3)
C4—C6—C7—C1	1.3 (3)	N2—C16—C17—C18	176.83 (17)
C4—C6—C7—C8	178.3 (2)	C16—C17—C18—C19	0.9 (3)
O1—C1—C7—C6	177.3 (2)	C17—C18—C19—C20	0.0 (4)
C2—C1—C7—C6	−2.4 (3)	C18—C19—C20—C21	−0.7 (3)
O1—C1—C7—C8	0.3 (4)	C19—C20—C21—C16	0.4 (3)
C2—C1—C7—C8	−179.5 (2)	C17—C16—C21—C20	0.5 (3)
C6—C7—C8—O2	−158.7 (2)	N2—C16—C21—C20	−177.45 (17)
C1—C7—C8—O2	18.2 (3)	C9—C10—N1—C11	1.0 (3)
C6—C7—C8—C9	23.0 (3)	N2—C11—N1—C10	178.78 (16)
C1—C7—C8—C9	−160.0 (2)	C12—C11—N1—C10	−2.1 (3)
O2—C8—C9—C13	36.0 (3)	N1—C11—N2—N3	178.98 (17)
C7—C8—C9—C13	−145.7 (2)	C12—C11—N2—N3	−0.31 (19)
O2—C8—C9—C10	−139.2 (2)	N1—C11—N2—C16	−2.9 (3)
C7—C8—C9—C10	39.1 (3)	C12—C11—N2—C16	177.79 (16)
C13—C9—C10—N1	1.3 (3)	C21—C16—N2—C11	−19.2 (3)
C8—C9—C10—N1	176.53 (18)	C17—C16—N2—C11	162.90 (18)
N1—C11—C12—C13	0.8 (3)	C21—C16—N2—N3	158.80 (17)
N2—C11—C12—C13	−179.92 (15)	C17—C16—N2—N3	−19.1 (2)
N1—C11—C12—C14	−179.01 (17)	C12—C14—N3—N2	0.0 (2)
N2—C11—C12—C14	0.27 (19)	C15—C14—N3—N2	179.44 (17)
C10—C9—C13—C12	−2.6 (3)	C11—N2—N3—C14	0.23 (19)
C8—C9—C13—C12	−177.90 (16)	C16—N2—N3—C14	−178.13 (14)

### Hydrogen-bond geometry (Å, º)

Cg3 and Cg4 are the centroids of rings C1–C4/C6/C7 and C16–C21, respectively.

**Table:**

*D*—H···*A*	*D*—H	H···*A*	*D*···*A*	*D*—H···*A*
O1—H1···O2	0.82	1.91	2.613 (2)	143
C21—H21···N1	0.93	2.41	3.019 (3)	123
C5—H5*A*···*Cg*3^i^	0.96	2.97	3.703 (3)	134
C20—H20···*Cg*4^i^	0.93	2.80	3.608 (2)	146

Symmetry code: (i) *x*, −*y*+1/2, *z*−1/2.
