# Supplementary figures and images for: Crystal structure of (2-hy­droxy-5-methyl­phen­yl)(3-methyl-1-phenyl-1H-pyrazolo­[3,4-b]pyridin-5-yl)methanone
Source: Acta Crystallogr E Crystallogr Commun. 2015 Jun 24;71(Pt 7):o501–2. doi: 10.1107/S2056989015011597 (PMC4518926; doi:10.1107/S2056989015011597)

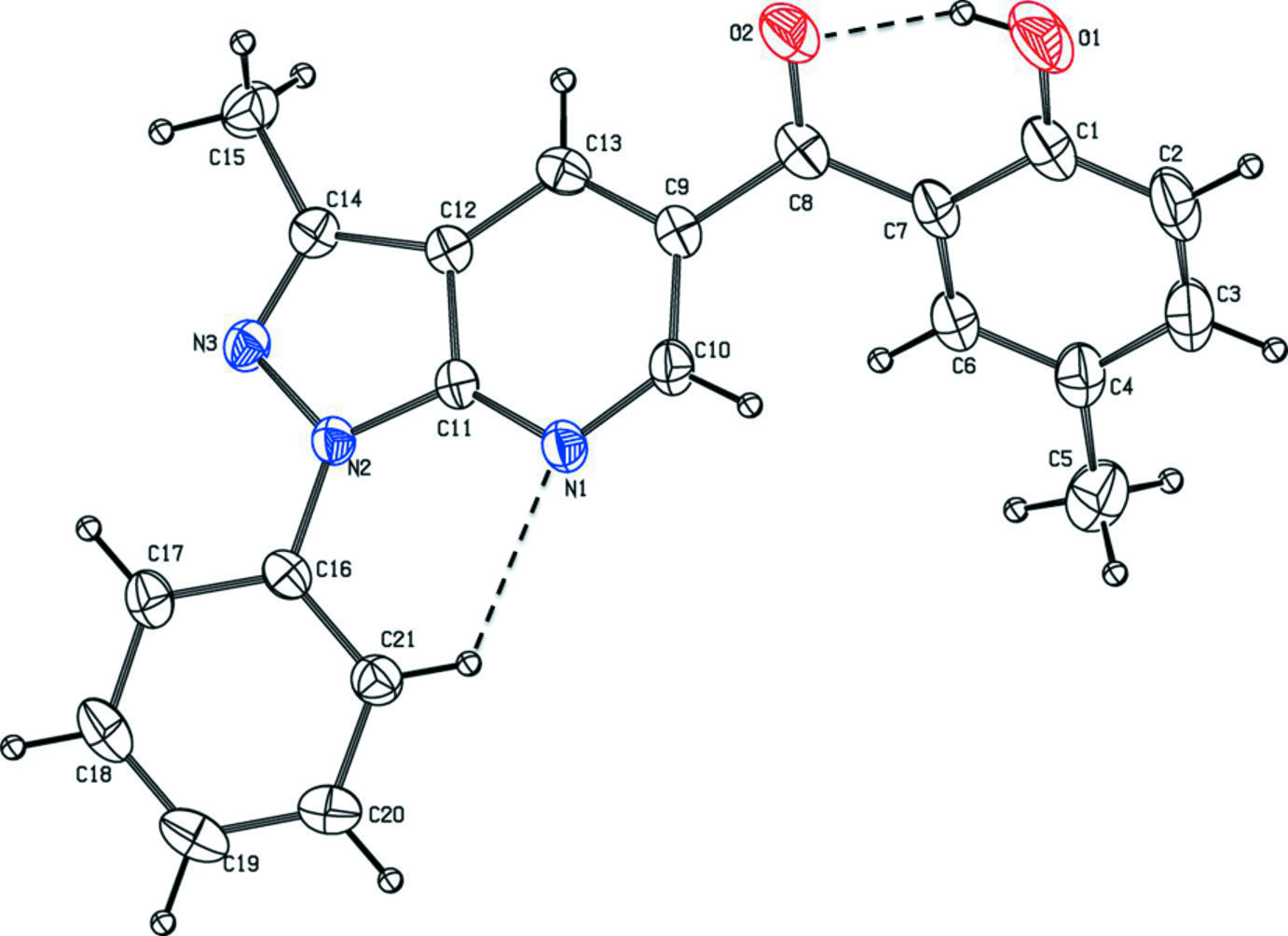

Supplement: Supplementary file 4 [file e-71-0o501-fig1.tif]

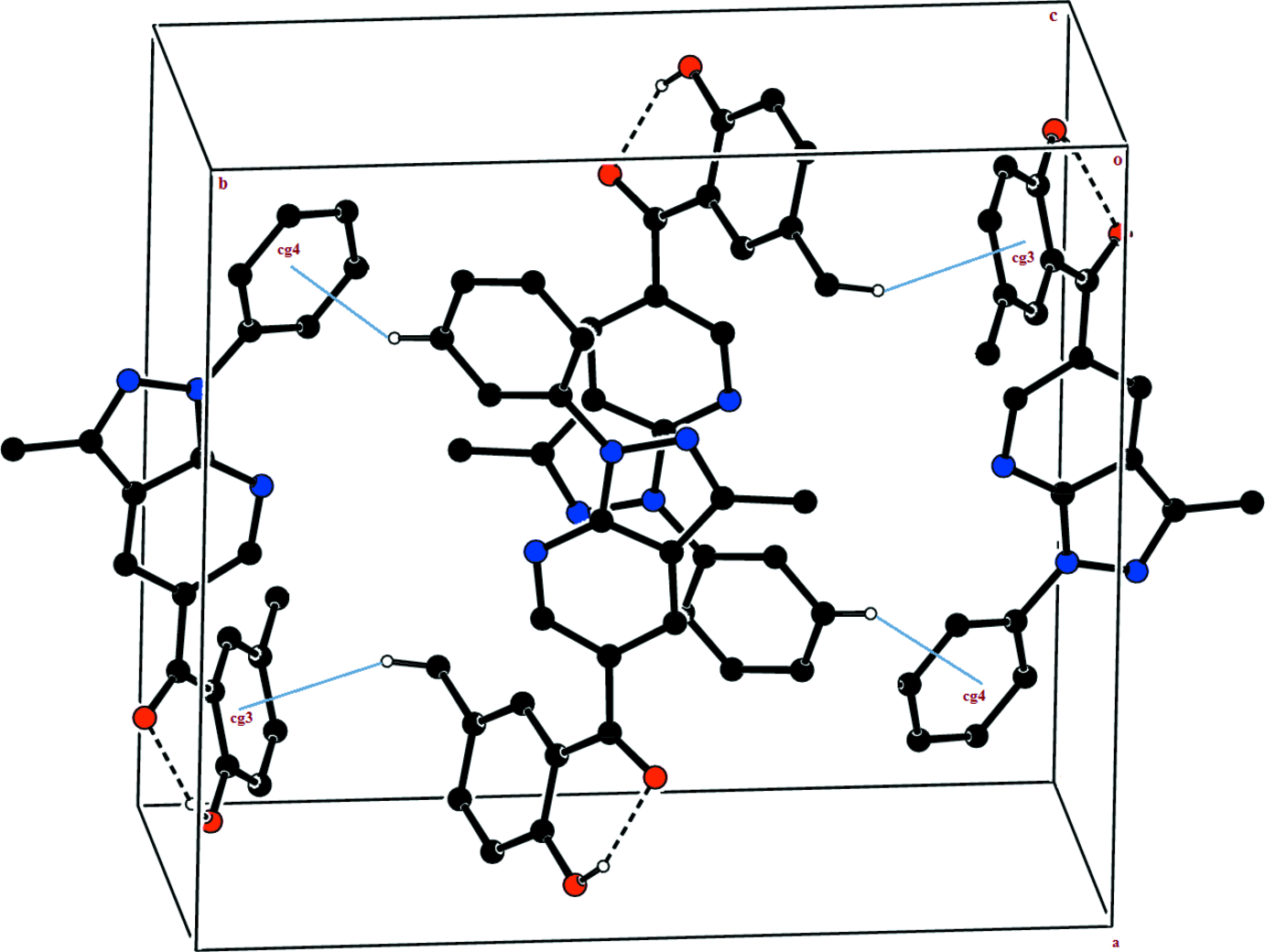

Supplement: Supplementary file 5 [file e-71-0o501-fig2.tif]
